# Supplementary material for: Screening for Glucose Metabolism Disorders, Assessment the Disse Insulin Resistance Index and Hospital Prognosis of Coronary Artery Bypass Surgery
Source: J Pers Med. 2021 Aug 17;11(8):802. doi: 10.3390/jpm11080802 (PMC8401009; doi:10.3390/jpm11080802)
Supplement: Supplementary file 1 [file jpm-11-00802-s001.zip › jpm-1313344-supplementary.pdf]

Supplementary Table S1. Multiple linear regression (Forward) in assessing the FFA relationship with other

Indicators (Model Summary).

Model	R	R^2	Adjusted R Square	Std. Error of the Estimate
1	0.374[a]	0.140	0.135	0.27717
2	0.427[b]	0.182	0.173	0.27101
3	0.463[c]	0.215	0.202	0.26622
4	0.494[d]	0.244	0.228	0.26191
5	0.515[e]	0.265	0.245	0.25899

a. Predictors: (Constant). TG

b. Predictors: (Constant). TG, HeartRate/

c. Predictors: (Constant). TG, HeartRate. APTT/

d. Predictors: (Constant). TG, HeartRate. APTT, BMI

e. Predictors: (Constant). TG, HeartRate. APTT, BMI, Glucose.

FFA – free fatty acids, TG – triglycerides, APTT – complex activated partial thromboplastin time, BMI – body mass index.

Supplementary Table S2. Multiple linear regression (Forward) in assessing the Disse index relationship with other indicators (Model Summary).

Model	R	R^2	Adjusted R Square	Std. Error of the Estimate
1	0.311[a]	0.096	0.078	9.63171
2	0.423[b]	0.179	0.144	9.27802

a. Predictors: (Constant). IVS

b. Predictors: (Constant). IVS. LVEF

IVS – interventricular septum. LV – left ventricular ejection fraction.

Supplementary Table S3. Predictors of the combined endpoint (postoperative complications or length of stay> 10 days) in binary logistic regression (Forward LR analysis, Model Summary).

Step	-2 Log likelihood	Cox & Snell R Square	Nagelkerke R Square
1	40.943[a]	0.340	0.463
2	35.474[a]	0.416	0.566
3	30.357[b]	0.479	0.652
4	24.174[b]	0.546	0.743

a. Estimation terminated at iteration number 6 because parameter estimates changed by less than .001.

b. Estimation terminated at iteration number 7 because parameter estimates changed by less than .001.

Supplementary Table S4. Metabolic predictors of the combined endpoint (poor outcome or length of stay> 10 days) in binary logistic regression (Forward LR analysis, Model Summary).

Step	-2 Log likelihood	Cox & Snell R Square	Nagelkerke R Square
1	166.022[a]	0.076	0.114
2	155.298[a]	0.135	0.202
3	148.160[a]	0.172	0.258

a. Estimation terminated at iteration number 5 because parameter estimates changed by less than .001.
